# Porous Ruthenium–Tungsten–Zinc Nanocages for Efficient Electrocatalytic Hydrogen Oxidation Reaction in Alkali

**DOI:** 10.3390/nano14090808

**Published:** 2024-05-06

**Authors:** Xiandi Sun, Zhiyuan Cheng, Hang Liu, Siyu Chen, Ya-Rong Zheng

**Affiliations:** Anhui Province Key Laboratory of Value-Added Catalytic Conversion and Reaction Engineering, Anhui Province Engineering Research Center of Flexible and Intelligent Materials, School of Chemistry and Chemical Engineering, Hefei University of Technology, Hefei 230009, China; zhiyuanc@mail.hfut.edu.cn (Z.C.); hangliu@mail.hfut.edu.cn (H.L.); chensy@mail.hfut.edu.cn (S.C.); yrzh@hfut.edu.cn (Y.-R.Z.)

**Keywords:** anion-exchange membrane fuel cell, hydrogen oxidation reaction, metal–organic frameworks, porous nanocages

## Abstract

With the rapid development of anion exchange membrane technology and the availability of high-performance non-noble metal cathode catalysts in alkaline media, the commercialization of anion exchange membrane fuel cells has become feasible. Currently, anode materials for alkaline anion-exchange membrane fuel cells still rely on platinum-based catalysts, posing a challenge to the development of efficient low-Pt or Pt-free catalysts. Low-cost ruthenium-based anodes are being considered as alternatives to platinum. However, they still suffer from stability issues and strong oxophilicity. Here, we employ a metal–organic framework compound as a template to construct three-dimensional porous ruthenium–tungsten–zinc nanocages via solvothermal and high-temperature pyrolysis methods. The experimental results demonstrate that this porous ruthenium–tungsten–zinc nanocage with an electrochemical surface area of 116 m^2^ g^−1^ exhibits excellent catalytic activity for hydrogen oxidation reaction in alkali, with a kinetic density 1.82 times and a mass activity 8.18 times higher than that of commercial Pt/C, and a good catalytic stability, showing no obvious degradation of the current density after continuous operation for 10,000 s. These findings suggest that the developed catalyst holds promise for use in alkaline anion-exchange membrane fuel cells.

## 1. Introduction

In recent years, hydrogen energy has received extensive attention as a potential alter-native to fossil fuels due to its high energy density of up to 140 MJ Kg^−1^, as well as its clean and environmentally friendly characteristics [1]. Hydrogen fuel cells are known for their high energy conversion efficiency and zero-carbon emissions, making them one of the most competitive technologies for hydrogen utilization [2]. Fuel cells are classified into five categories based on the type of electrolyte and the operating conditions, including proton exchange membrane fuel cells (PEMFCs), anion exchange membrane fuel cells (AEMFC), phosphoric acid fuel cells, molten carbonate fuel cells, and solid oxide fuel cells [3]. So far, PEMFCs have been at the forefront of cutting-edge energy conversion technologies used in various applications, such as automobiles, communications, and aerospace. However, PEMFCs require platinum (Pt)-based electrocatalysts, acid-resistant electric pile hardware, and perfluorinated membranes, resulting in high production costs [4]. The AEMFC represents a highly efficient and environmentally friendly power generation technology. It is regarded as a new-generation energy power system due to its high cell efficiency, excellent catalyst stability, and flexible fuel selectivity. Anion-exchange membranes are seen as the next generation of cost-effective fuel cells because they can use efficient, non-precious metal catalysts in the cathodic oxygen reduction reaction, significantly reducing fuel cell costs [5]. However, the kinetics of Pt group metals required for anode hydrogen oxidation reactions (HORs) in an alkaline media are 2–3 orders of magnitude lower than those in an acidic media. Therefore, improving the performance of noble metal catalysts or developing efficient low-cost alkaline HOR catalysts remains a major challenge [6,7].

Ruthenium (Ru) possesses a similar hydrogen binding energy to Pt, but is much less expensive than Pt, making it a potential substitute to Pt for alkaline HORs [8,9]. However, Ru exhibits oxyphilic properties at anodic potentials above 0.1 V compared to a reversible hydrogen electrode (RHE), leading to a decreased electrochemical HOR performance [10]. Several strategies have recently been developed to enhance the performance of Ru-based catalysts, including compositional effects [11,12,13], carrier effects [14,15,16,17], size effects [18,19], and structural effects [20,21]. In the case of loaded catalysts, they are usually overlooked when the particles are loaded or embedded in the carrier, which may result in underutilization of the active sites [22,23].

Metal–organic frameworks (MOFs), three-dimensional ordered porous structure materials, have garnered significant attention due to their high specific surface area and controllable porous structure [24]. Furthermore, MOF structures offer the advantages of being easily designed and modified, enabling the direct synthesis of various functional materials derived from MOFs. MOF-derived catalysts often possess hollow structures, facilitating mass transfer and intermediate diffusion, thus improving catalytic performance [25,26,27,28]. For example, Ma et al. [29] used octahedral zeolitic imidazolate framework-8 (ZIF-8) as a template to construct sub-2 nm RuMo-anchored hollow carbon catalysts, exhibiting a high activity for alkaline HOR, with a mass activity of 3.83 A mg_Ru_^−1^, which is 25 times higher than that of commercial Ru/C. Qiu et al. [30] reported a general method for synthesizing binary, ternary, and high-entropy nanoparticles using a 2D MOF-assisted pyrolysis-replacement alloying technique. The developed Co_0.2_Ru_0.7_Pt_0.1_/PNC nanosheets exhibited a mass activity as high as 1.84 A mg^−1^ at an overpotential of 50 mV. Therefore, constructing high surface area catalysts and improving their exposure rates are the key factors for enhancing the alkaline HOR performance.

Here, we synthesize a three-dimensional hollow porous ruthenium–tungsten–zinc (Ru-W-Zn-O) nanocage through a hydrothermal reaction and high-temperature pyrolysis processes. The optimized Ru_1_W_0.14_Zn_1.47_O_y_ catalyst exhibits an enhanced HOR performance in alkaline conditions compared to commercial Pt/C and Ru/C. Specifically, the kinetic current densities (*j*_k_) were 1.82 and 1.04 times higher than those of commercial Pt/C and Ru/C, respectively. After a constant voltage test for 10,000 s, the current density of Ru_1_W_0.14_Zn_1.47_O_x_ shows no significant decay. These fundings make Ru_1_W_0.14_Zn_1.47_O_x_ a promising candidate for alkaline AEMFC anode materials.

## 2. Materials and Methods

### 2.1. Materials

Zn (NO_3_)_2_·6H_2_O, cetyltrimethylammonium bromide (CTAB), potassium hydroxide (KOH), isopropanol, and methanol were purchased from Sinopharm (Shanghai, China), and 2-methyl imidazole was purchased from Beijing Jin Ming Biotechnology Co., Ltd. (Beijing, China). RuCl_3_·xH_2_O, Ru/C (20 wt%), and Na_2_WO_4_ were purchased from Macklin (Shanghai, China). Pt/C (20 wt%) was purchased from Suzhou Sinero Technology Co., Ltd. (Suzhou, China). Nafion^®^117 solution was purchased from Sigma-Aldrich (St. Louis, MO, USA) (~5% in a mixture of lower aliphatic alcohols and water, containing 15~20% water). All reagents were analytically pure and used as received without further purification.

### 2.2. Material Characterization

Transmission electron microscopy (TEM) and Field-Emission Scanning Electron Microscopy (SEM) images were obtained from a Hitachi H7700 and SU8020 (Tokyo, Japan) at an accelerating voltage of 100 KV. The data of high-revolution TEM images and EDS elemental mapping were obtained from a JEM-2100F (Tokyo, Japan). The catalyst solution was applied onto 300-mesh copper grids coated with formvar/carbon support film (Beijing Zhongjing Key Technology Co., Ltd., Beijing, China). The powder X-ray diffraction (XRD) patterns were obtained on Philips X’Pert Pro Super with a Cu Ka radiation source (λ = 1.541841 Å). X-ray photoelectron spectra (XPS) were measured using an Al Kα radiation source on a Thermo Fisher ESCALAB 250Xi (Waltham, MA, USA). The peak shifts caused by apparent charging were calibrated using the carbon C 1*s* peak set to 284.8 eV. All spectra were collected in ambient conditions. All electrochemical performances were conducted with an electrochemical workstation (Autolab, Metrohm, Herisau, Switzerland). The noble metal mass content of Ru in Ru_1_W_0.14_Zn_1.47_O_x_ and RuZnO_x_ nanocages were determined by inductively coupled plasma-Mass Spectrometry (ICP-MS, Agilent Technologies 7500 series, Santa Clara, CA, USA).

### 2.3. Methods

#### 2.3.1. Preparation of ZIF-8

In a typical preparation, 1.22 mmol of Zn (NO_3_)_2_·6H_2_O was dissolved in 12.5 mL of deionized water and stirred at 350 rpm. Separately, 70.64 mmol of 2-methyl imidazole and 1.22 mmol of CTAB were mixed with 87.5 mL deionized water. The Zn (NO_3_)_2_·6H_2_O solution was then quickly injected into the second solution and stirred continuously for 5 min. The above operations were conducted at room temperature, followed by standing for several hours to obtain a uniformly dispersed ZIF-8 solution.

#### 2.3.2. Synthesis of Ru_1_W_0.14_Zn_1.47_O_x_ Nanocage

As is typical for the synthesis of nanocages, 8 mL of the white ZIF-8 solution was taken for centrifugation, and the precipitate was rinsed with methanol several times. Then, 3 mL of deionized water was added to the precipitate and dispersed by ultrasound. Meanwhile, 10 mg of RuCl_3_·xH_2_O and 2 mg of Na_2_WO_4_ were dispersed in a mixed solution of 2 mL deionized water and 1 mL methanol. The resulting solution was then injected into the ZIF-8 suspension and thoroughly mixed by stirring for 30 min. After that, the mixture was transferred into a 20 mL Teflon-lined autoclave and heated at 80 °C for 2 h. Finally, the resulting sample (denoted as RuW-2@ZIF-8) was then placed in a porcelain boat and calcined in a reducing gas, H_2_/Ar (95%). The temperature was raised to 300 °C at a heating rate of 10 °C/min and maintained for 2 h. After cooling to room temperature, the Ru_1_W_0.14_Zn_1.47_O_x_ nanocage was finally obtained. Soon afterwards, using 6 mg of W precursor while Ru was unchanged (denoted as RuW-6@ZIF-8) as the precatalyst, the Ru_1_W_0.33_Zn_1.14_O_x_ nanocage could be obtained. The catalyst RuZn_x_O_y_ was obtained without using W precursor and Ru was kept unchanged. The atomic ratio between Ru, W, and Zn was obtained based on the XPS analysis.

#### 2.3.3. Electrochemical Measurements

As is generally carried out for electrochemical measurements, 1 mg of the developed catalyst was added to 400 μL isopropanol, while 1 mg of carbon black was added to 390 μL of isopropanol and 10 μL of Nafion (5 wt%) solution. The mixture was continuously ultrasonicated for several hours until a homogeneous ink formed. Then, 15 μL of catalyst ink was evenly pipetted onto the glassy carbon working electrode. The catalyst loading on the working electrode was 0.19 mg/cm^2^.

Electrochemical tests were performed using a three-electrode system. The working electrode was a rotating disk electrode with a disk area of 0.196 cm^2^ and a rotation speed ranging from 400 to 2500 rpm. A saturated calomel electrode (SCE) and a graphite carbon electrode were used as the reference electrode and the counter electrode, respectively. Prior to testing, the 0.1 M KOH electrolyte was saturated with H_2_. The catalysts were first tested using cyclic voltammetry (CV) followed by linear sweep voltammetry (LSV) to ensure a quick attainment of steady state. The voltage range of CV was −0.05~0.2 V vs. RHE, with a scan rate of 10 mV s ^−1^. For LSV, the scan rate was 1 mV s ^−1^, and the rotation speed was 1600 rpm. The stability test of Ru_1_W_0.14_Zn_1.47_O_x_ was performed using a chronoamperometry test (CA) at a constant potential of 50 mV vs. RHE in H_2_-saturated 0.1 M KOH. Unless otherwise mentioned, the potentials in this work were converted to the reversible hydrogen electrode by the equation:

$$E_{RHE}=E_{SCE}+0.059\times pH+0.253$$

Due to the fact that Ru-based materials tend to absorb OH* in the hydrogen underpotential deposition (UPD) area. In this work, the Cu UPD method was employed to determine the electrochemical active surface areas (ECSAs) of Ru-based catalysts. The catalysts were subjected to multiple cycles of scanning, ranging from 0 to 0.7 V vs. RHE in order to obtain a stable CV curve as a background measurement in an Ar-saturated 0.1 M H₂SO₄ solution, with a scan rate of 10 mV s^−1^. When the deposition of Cu occurs at 0.3 V vs. RHE for a duration of 100 s, the stripping of Cu-UPD commences at potentials ranging from 0.3–0.7 V vs. RHE in an Ar-saturated 0.1M H_2_SO_4_ solution containing 2 mM CuSO_4_, with a scanning rate of 10 mV s^−1^. The value of ECSA (cm^2^ g^−1^) can be calculated using the equation:

$$ECSA(\frac{{cm}_{metal}^{2}}{g_{metal}})=\frac{Q_{Cu}}{m_{metal}\cdot 420\;\mu C\;{cm}^{-2}}$$

where *Q*_Cu_ is the measured integral charge, *m*_metal_ is the mass loading of metals on the electrode, 420 μC cm^−2^_metal_ is the surface charge density which is assumed for a monolayer adsorption of Cu-UPD on metal.

After LSV test, electrochemical impedance spectroscopy (EIS) was performed over a frequency range from 200 kHz to 0.1 kHz with a voltage perturbation of 10 mV. The potential after *iR* correction is calculated using the equation:

$$E_{iR}=E-iR$$

where *E* and *R* correspond to the measured potential and the solution resistance, respectively. The kinetic current density (*j*_k_) can be obtained by the Koutecky–Levich equation:

$$\frac{1}{j}=\frac{1}{j_{k}}+\frac{1}{j_{d}}=\frac{1}{j_{k}}+\frac{1}{Bc_{0}w^{1/2}}$$

where *j*, *j*_d_, *B*, *c*_0_, and *ω* represent the measured current, the diffusional current, the Levich constant, the solubility of H_2_ (7.33 × 10^−4^ mol L^−1^), and the rotating speed, respectively. The exchange current density (*j*_0_) could be obtained by the Butler–Volmer (B-V) equation:

$$j_{k}=j_{0}\left[e^{\frac{\alpha F}{RT}\eta }-e^{\frac{-\left(1-\alpha \right)F}{RT}\eta }\right]$$

where *α*, *η*, *R*, *T*, and *F* represent the transfer coefficient, the overpotential, the molar gas constant (8.314 J mol^−1^ K^−1^), the operating temperature, and the Faraday constant, respectively. *j*_0_ could be also obtained from the approximate B-V equation:

$$j=j_{0}\frac{\eta F}{RT}$$

## 3. Results and Discussion

### 3.1. Catalyst Synthesis and Characterization

**Figure 1:**
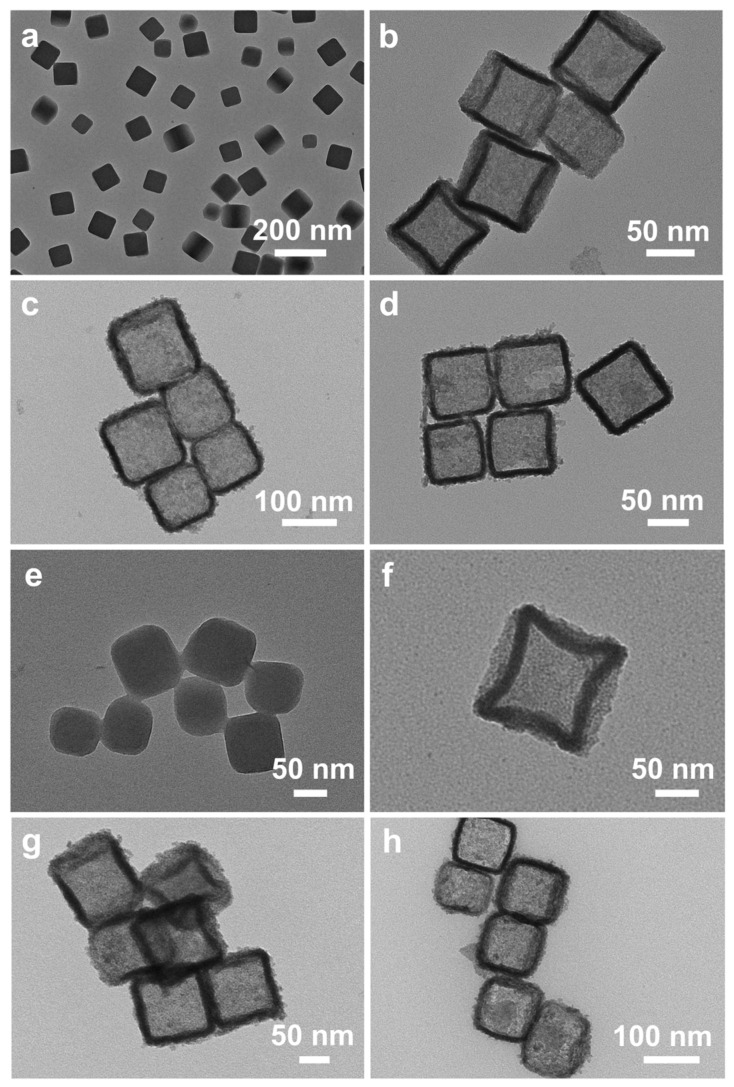
Morphology characterization. (**a**–**d**) TEM images of as-prepared (**a**) ZIF-8, precatalysts of (**b**) Ru@ZIF-8, (**c**) RuW-2@ZIF-8, and (**d**) RuW-6@ZIF-8, respectively. (**e**,**f**) TEM images of (**e**) ZIF-8 after annealing, (**f**) RuZn_x_O_y_, (**g**) Ru_1_W_0.14_Zn_1.47_O_x_, and (**h**) Ru_1_W_0.33_Zn_1.14_O_x_, respectively.

**Figure 2:**
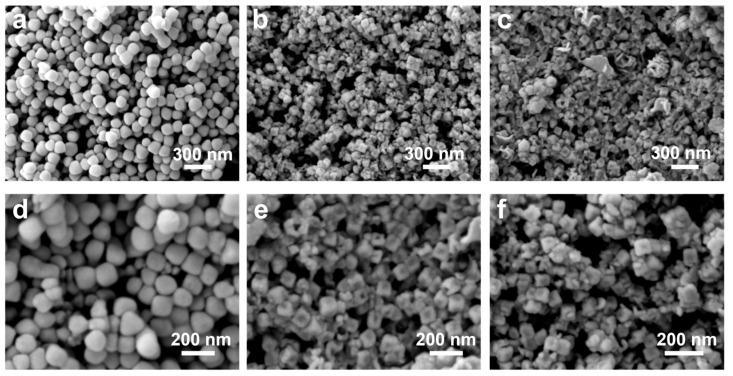
SEM images of (**a**,**d**) ZIF-8 after annealing, (**b**,**e**) Ru_1_W_0.14_Zn_1.47_O_x_, and (**c**,**f**) Ru_1_W_0.33_Zn_1.14_O_x_.

We fabricated the Ru_1_W_0.14_Zn_1.47_O_x_ nanocage through a two-step process involving hydrothermal and calcination treatments. ZIF-8 was first prepared as the template as detailed in the Methods section. The morphology of the pure ZIF-8 was imaged by TEM, showing a cubic structure with an average size of approximately 100 nm. Furthermore, the TEM images demonstrate that ZIF-8 was uniformly distributed on the copper grid without any agglomeration (Figure 1a). To prepare the Ru-W-Zn-O nanocomposites, RuCl_3_·xH_2_O and Na_2_WO_4_ were used as the Ru and W precursors, respectively. Different ratios of Ru/W were applied during the hydrothermal reaction to control the compositions. After introducing Ru or W ions, ZIF-8 underwent hydrolysis during the hydrothermal treatment, leading to the formation of the hollow nanocage structure, as shown in Figure 1b–d, with Ru and W confined in the substrate. Compared to Ru@ZIF-8 (Figure 1b), the relatively lighter contrast of the hollow structures of RuW@ZIF-8 (Figure 1c,d), indicating the introduction of W might accelerate the replacement of Zn and creating a favorable structure for further electrochemical investigation. To obtain the metallic Ru, which was crucial for electrocatalytic HOR, the precatalysts were subsequently annealed at 300 °C under Ar/H_2_ (5%) atmosphere and kept at this temperature for 2 h. The atomic ratios of the Ru-W-Zn-O catalysts after annealing were confirmed by XPS analysis. After annealing, ZIF-8 still shows the cubic structure (Figure 1e). In contrast, RuZn_x_O_y_, Ru_1_W_0.14_Zn_1.47_O_x_, and Ru_1_W_0.33_Zn_1.14_O_x_ catalysts corresponding to Ru@ZIF-8, RuW-2@ZIF8, and RuW-6@ZIF-8 precatalysts, respectively, maintain the pristine hollow structure without any aggregations or collapses. Moreover, the high-temperature treatment also generated pores on the nanocages, which could facilitate H_2_ transfer and improve the electrocatalytic performance (Figure 1f,g). The SEM images of the annealed samples at different magnifications also demonstrated that the catalysts retained their cubic structure (Figure 2a–f).

**Figure 3:**
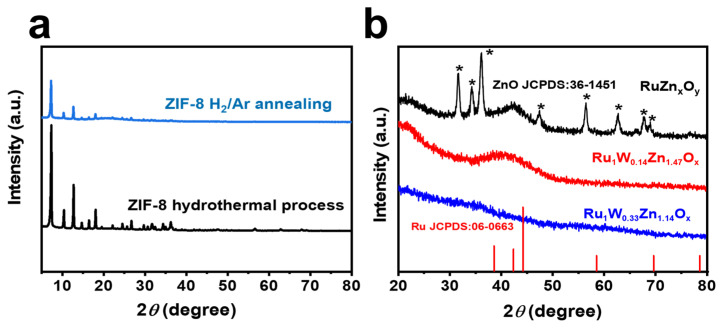
XRD patterns of (**a**) ZIF-8 with different treatment ways, (**b**) RuZn_x_O_y_, Ru_1_W_0.33_Zn_1.14_O_x_ and Ru_1_W_0.14_Zn_1.47_O_x_.

We further conducted XRD characterization to investigate the phase structures of the catalysts. After hydrothermal and annealing treatments, ZIF-8 retained its pristine structure (Figure 3a). The XRD pattern of RuZn_x_O_y_ reveals the presence of ZnO impurity and the hexagonal close-packed Ru (*h*-Ru) phase (JCPDS No. 06-0663). In contrast, after the induction of W, the peaks of ZnO impurities are absent, indicating that Ru-W-Zn-O composites tend to exhibit amorphous structures (Figure 3b). The Ru_1_W_0.14_Zn_1.47_O_x_ catalyst shows a broad peak at approximately 42°, which could be assigned to *h*-Ru. However, with increasing W content, the characteristic peaks of *h*-Ru disappear and obvious diffraction peaks of tungsten oxide are observed, which could be attributed to the highly dispersion of W within the nanocomposites.

**Figure 4:**
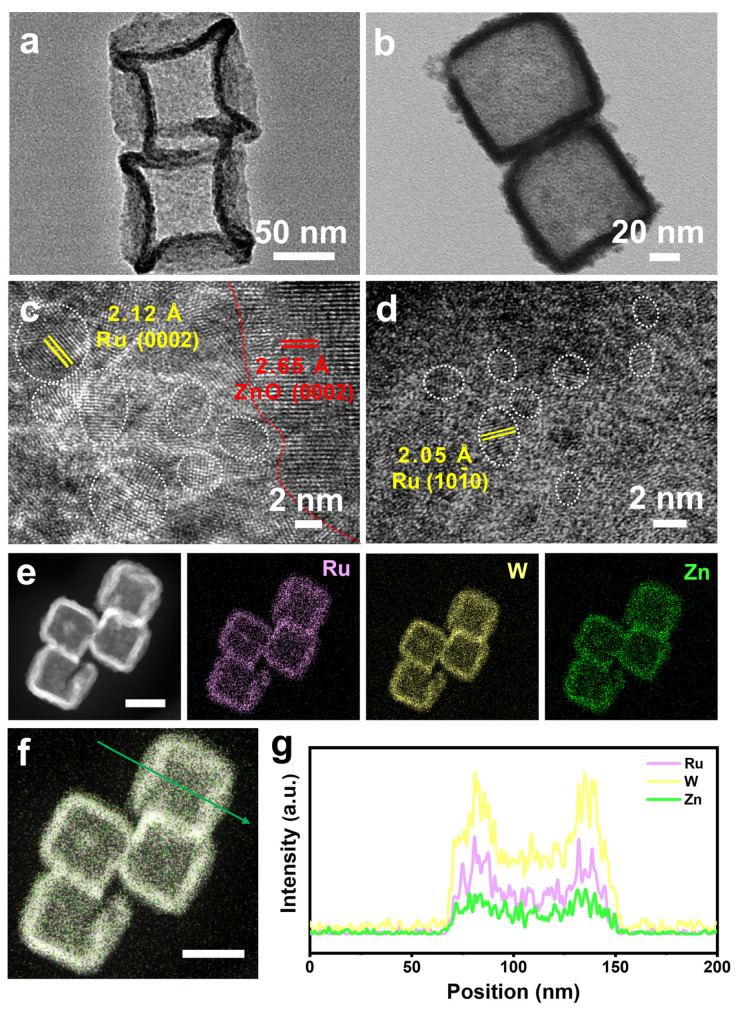
TEM images of (**a**) RuZn_x_O_y_ and (**b**) Ru_1_W_0.14_Zn_1.47_O_x_, respectively. HRTEM images of (**c**) RuZn_x_O_y_ and (**d**) Ru_1_W_0.14_Zn_1.47_O_x_, respectively. (**e**) The EDS elemental mapping analysis of Ru_1_W_0.14_Zn_1.47_O_x_. Scale bar: 50 nm. (**f**,**g**) EDX line-scanning profile of Ru_1_W_0.14_Zn_1.47_O_x_ catalyst. Scale bar: 50 nm.

The high-resolution TEM (HRTEM) images were employed to investigate the atomic lattice of the catalysts. Figure 4a,b show that both RuZn_x_O_y_ and Ru_1_W_0.14_Zn_1.47_O_x_ nanocages retain a size of approximately 100 nm. These nanocages are composed of numerous nanoparticles and exhibit porous structures. In the HRTEM image of RuZn_x_O_y_ (Figure 4c), the lattice distances of 2.12 Å and 2.65 Å are ascribed to h-Ru (0002) and ZnO (0002) facets, respectively. Ru nanoparticles, with a size ranging from 2 to 5 nm, are seen to be well distributed on the substrate. However, continuous lattice fringes of ZnO are absent from the Ru_1_W_0.14_Zn_1.47_O_x_ catalyst after the introduction of W (Figure 4d), and the size of the Ru nanoparticles decreased to approximately 1~2 nm, which is consistent with the XRD results. Additionally, EDS elemental mapping and line-scanning analysis (Figure 4e–g) demonstrate that Ru, W, and Zn are evenly distributed in the nanocages.

**Figure 5:**
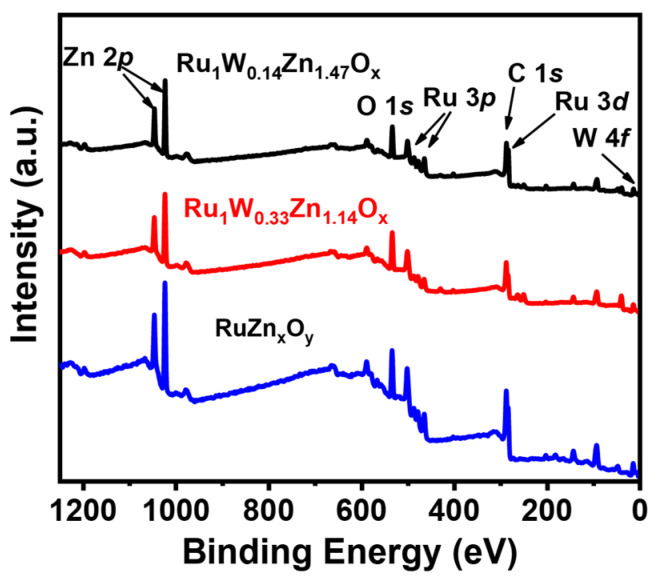
XPS survey spectra of RuZn_x_O_y_, Ru_1_W_0.14_Zn_1.47_O_x_, and Ru_1_W_0.33_Zn_1.14_O_x_.

**Figure 6:**
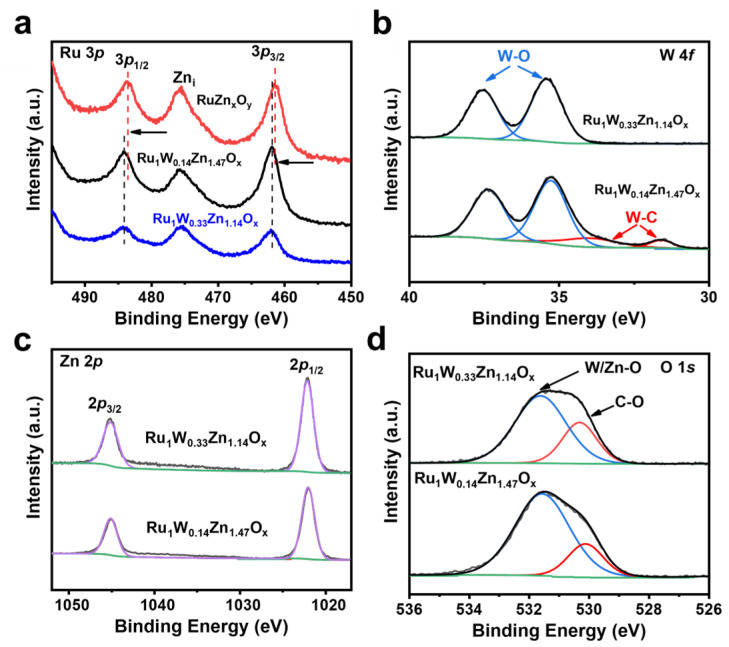
High-resolution XPS spectra of (**a**) Ru 3*p* of RuZn_x_O_y_, Ru_1_W_0.14_Zn_1.47_O_x_ and Ru_1_W_0.33_Zn_1.14_O_x_. High-resolution XPS spectra of (**b**) W 4*f*, (**c**) Zn 2*p*, and (**d**) O 1*s* of Ru_1_W_0.14_Zn_1.47_O_x_. and Ru_1_W_0.33_Zn_1.14_O_x_, respectively.

We further conducted XPS analysis to characterize the composition and oxidation state of the catalyst. The XPS survey spectrum of RuZn_x_O_y_ indicates the presence of Ru, Zn, C, and O elements. Ru_1_W_0.14_Zn_1.47_O_x_ and Ru_1_W_0.33_Zn_1.14_O_x_ exhibit an additional characteristic peak of W compared to RuZn_x_O_y_, indicating successful W and Ru doping (Figure 5). The Ru 3*p* was selected to investigate the valence state of Ru since the binding energies of Ru 3*d* overlap with those C 1*s* [31]. The high-resolution XPS spectra of Ru 3*p* of RuZn_x_O_y_ and Ru_1_W_0.14_Zn_1.47_O_x_ after annealing in H_2_/Ar atmosphere (Figure 6a) exhibit two peaks at 461.69 and 461.38 eV, corresponding to metallic Ru [32]. The binding energy of the Ru 3*p* in the Ru_1_W_0.14_Zn_1.47_O_x_ catalyst is positively shifted compared to RuZn_x_O_y_, indicating a modification of the electronic structure of Ru due to the introduction of W. The electron transfer between the active site of the Ru and other atoms can modulate the d-band structure of Ru and optimize the binding ability between H* and Ru, thus enhancing the HOR performance. The high-resolution W 4*f* spectrum of Ru_1_W_0.14_Zn_1.47_O_x_ (Figure 6b) shows peaks at 37.31 eV and 35.27 eV corresponding to W-O, and at 33.94 eV and 31.54 eV corresponding to W-C [32]. However, Ru_1_W_0.33_Zn_1.14_O_x_ only shows peaks at 37.63 and 35.4 eV corresponding to W-O. As shown in Figure 6c, the peaks at 1021.94 and 1022.15 eV corresponding to Zn 2*p*_3/2_, and those at 1044.86 and 1045.19 eV corresponding to Zn 2*p*_1/2_, indicating the presence of Zn^2+^. Figure 6d shows that the peaks located at 531.45 and 531.71 eV corresponding to C=O, and those at 530.02 and 530.40 eV corresponding to W/Zn-O, demonstrate the formation of oxidized W and Zn [33]. These results confirm that Ru remains the metallic state in the developed RuZn_x_O_y_, Ru_1_W_0.14_Zn_1.47_O_x_, and Ru_1_W_0.33_Zn_1.14_O_x_ catalysts, and the slight shift in Ru 3*p* between RuZn_x_O_y_ and W-doped composites is attributed to the charge transfer effect, which can optimize the electronic structure of the active sites.

### 3.2. Electrochemical Characterization

**Figure 7:**
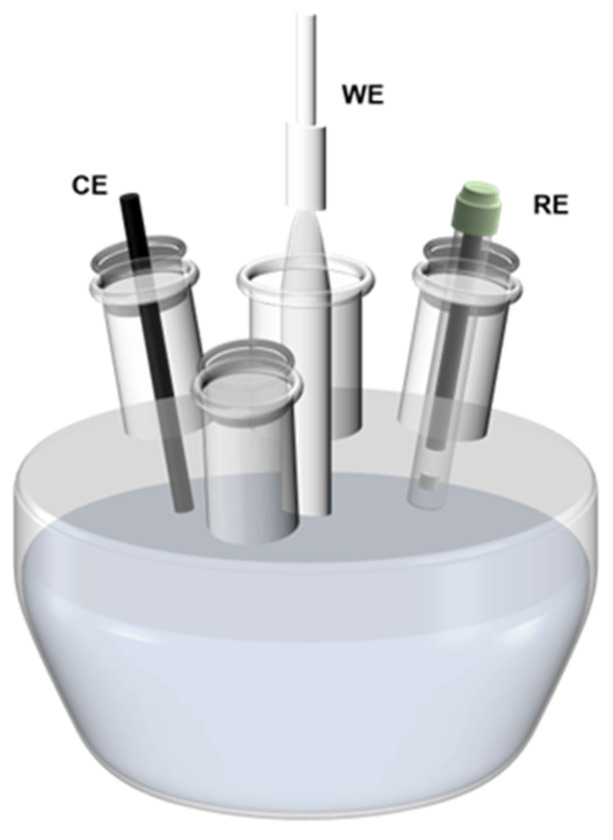
Schematic illustration of the three-electrode system.

The electrochemical HOR performances of the catalysts were evaluated using a rotating disk electrode in a three-electrode system (Figure 7) in H_2_-saturated 0.1 M KOH.

**Figure 8:**
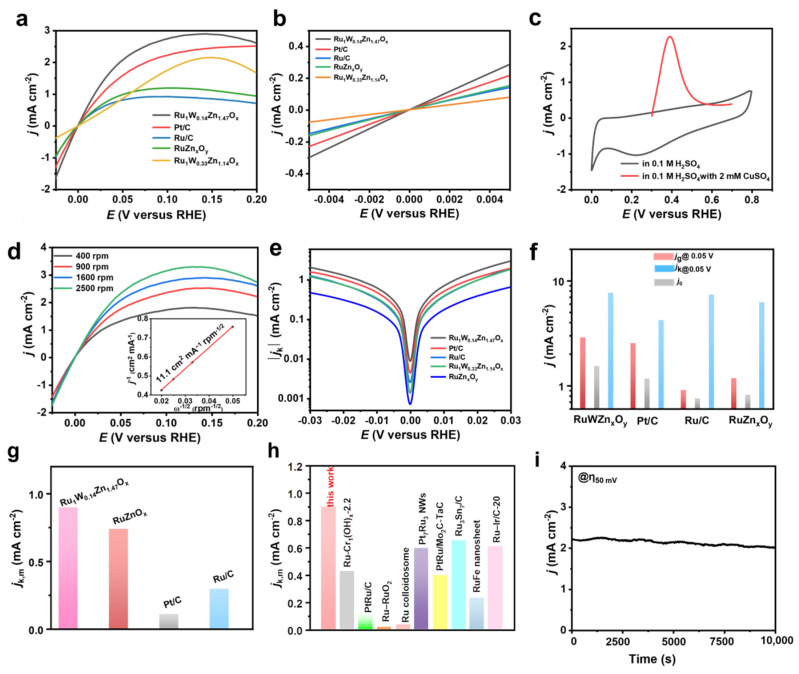
Electrocatalytic HOR performances. (**a**) Polarization curves of Ru_1_W_0.14_Zn_1.47_O_x_, Pt/C, Ru/C, RuZn_x_O_y_, and Ru_1_W_0.33_Zn_1.14_O_x_, respectively, in H_2_-saturated 0.1 M KOH solution. (**c**) The Cu_upd_ stripping voltammogram of Ru_1_W_0.14_Zn_1.47_O_x_. The loading of Ru is about 10 µg. (**b**) Micropolarization region between −5 to 5 mV vs. RHE of the corresponding catalysts. (**d**) Polarization curves of Ru_1_W_0.14_Zn_1.47_O_x_ at different rotation rates. The Koutecky–Levich plot of Ru_1_W_0.14_Zn_1.47_O_x_ at an overpotential of 50 mV vs. RHE inset of (**d**). (**e**) Tafel plots present the kinetic current densities of Ru_1_W_0.14_Zn_1.47_O_x_, Pt/C, Ru/C, RuZn_x_O_y_, and Ru_1_W_0.33_Zn_1.14_O_x_, respectively, derived from the Butler–Volmer equation fitting. (**f**) Comparison of the *j*_g_, *j*_k_, and *j*_0_ of Ru_1_W_0.14_Zn_1.47_O_x_, RuZn_x_O_y_, Pt/C, and Ru/C at 50 mV vs. RHE. (**g**) Comparison of mass normalized current density for Ru_1_W_0.14_Zn_1.47_O_x_, RuZnO_x_, commercial Pt/C, and Ru/C. (**h**) Comparison of the *j*_k,m_ at an overpotential of 50 mV vs. RHE for Ru_1_W_0.14_Zn_1.47_O_x_ in this work with other alkaline HOR catalysts. The column diagrams are duplicated from the literature: Ru-Cr_1_(OH)_x_-_2.2_ ([35]), PtRu/C ([36]), Ru–RuO_2_ ([37]), Ru colloidosome ([14]), Pt_7_Ru_3_ NWs ([12]), PtRu/Mo_2_C-TaC ([38]), Ru_3_Sn_7_/C ([5]), RuFe nanosheet ([39]), Ru–Ir/C-20 ([11]). (**i**) Chronoamperometry (*j*-*t*) response of Ru_1_W_0.14_Zn_1.47_O_x_ in H_2_-saturated 0.1 M KOH solution at 50 mV vs. RHE. The loading of Ru is 8.55 µg cm^−2^ in all electrocatalytic tests, except Cu-UPD testing.

The HOR polarization curves of the catalysts were obtained through LSV tests conducted at a rotating speed of 1600 rpm with a scan rate of 1 mV s^−1^. Figure 8a shows that the Ru_1_W_0.14_Zn_1.47_O_x_ catalyst exhibits a higher anode current density compared to RuZn_x_O_y_, commercial Ru/C, and Pt/C. For commercial Ru/C, the catalyst loading was adjusted to 0.5 mg cm^−2^ to prepare a well-dispersed catalyst membrane on the working electrode. The result suggests that the introduction of W is essential for enhancing HOR activity. However, the electrochemical performance decreases when there is an excessive amount of W, indicating that an excessive usage of W might cover the active sites of Ru and reduce the porosity of the composites, thus hindering H_2_ transfer and decreasing the electrochemical performance. The geometric current density (*j*_g_) of Ru_1_W_0.14_Zn_1.47_O_x_ increases rapidly at around 0 V vs. RHE compared to RuZn_x_O_y_, commercial Pt/C, and Ru/C, as confirmed by the micro-polarization region (−5 to 5 mV) analysis (Figure 8b). The exchange current density (*j*_0_) of Ru_1_W_0.14_Zn_1.47_O_x_ was calculated as 1.54 mA cm^−2^ in the micro-polarization region using the Butler–Volmer equation, which is 1.32 and 2.03 times higher than that of commercial Pt/C and Ru/C, respectively. The Cu-UPD analysis showed that the ECSA of Ru_1_W_0.14_Zn_1.47_O_x_ reached 116 m^2^ g^−1^ (Figure 8c). After normalization by ECSA, the specific activity (*j*_0,s_) of Ru_1_W_0.14_Zn_1.47_O_x_ was found to be 0.026 mA cm_ECSA_^−2^, close to that of the commercial Ru/C catalyst [34]. This suggests that the enhanced HOR activity of Ru_1_W_0.14_Zn_1.47_O_x_ can be attributed to the favorable structure created, which has abundant exposed active sites. The polarization curves of Ru_1_W_0.14_Zn_1.47_O_x_ at various rotation speeds, ranging from 400 to 2500 rpm, showing that the anodic current densities consistently increase with the higher rotation speeds (Figure 8d). The Koutecký–Levich plot at an overpotential of 50 mV reveals a linear relationship between *j*^−1^ and *ω*^−1/2^.

The slope of Ru_1_W_0.14_Zn_1.47_O_x_ is 11.1 cm^2^ mA^−1^ rpm^−1/2^, which is close to the theoretical value of 14.8 cm^2^ mA^−1^ rpm^−1/2^, confirming that the current is mainly derived from the HOR process involving a 2*e*^−^ transfer (inset of Figure 8d). The Tafel plots (Figure 8e) further demonstrate that the introduction of W significantly promotes the HOR process in alkali. The Ru_1_W_0.14_Zn_1.47_O_x_ catalyst exhibits the highest *j*_0_ and *j*_g_ at 50 mV vs. RHE compared to commercial Pt/C, Ru/C, and RuZn_x_O_y_, as shown in Figure 8f. Using the Koutecký–Levich equation, we obtained a geometric kinetic current density (*j*_k_) of 7.69 mA cm^−2^ for the Ru_1_W_0.14_Zn_1.47_O_x_ catalyst, which is 1.82 times higher than that of commercial Pt/C, and similar to that of Ru/C, despite the Ru/C loading being approximately 2.63 times higher than that of Ru_1_W_0.14_Zn_1.47_O_x_. The mass content of Ru in Ru_1_W_0.14_Zn_1.47_O_x_ and RuZnO_x_ nanocages has been determined to be approximately 4.5 wt% by ICP-MS. The mass activity (*j*_k,m_) was obtained by normalizing the kinetic current density with respect to the mass of noble metal. As shown in Figure 8g, the *j*_k,m_ of Ru_1_W_0.14_Zn_1.47_O_x_ catalyst is 0.9 mA µg^−1^ at 50 mV vs. RHE, which is 1.22, 8.18, and 3.04 times higher than that of RuZnO_x_, commercial Pt/C, and Ru/C, respectively. Furthermore, the value of *j*_k,m_ for the Ru_1_W_0.14_Zn_1.47_O_x_ catalyst indicates the enhanced catalytic activity compared to other reported HOR electrocatalysts in alkaline media (Figure 8h).

Besides HOR activity, operating stability is another important factor for application. Therefore, we conducted a stability test using chronoamperometry at a constant potential of 50 mV vs. RHE in H_2_-saturated 0.1 M KOH. Figure 8i shows that the HOR current density of the Ru_1_W_0.14_Zn_1.47_O_x_ catalyst remains stable without noticeable decay over 10,000 s. These results demonstrate the excellent HOR activity and stability of the Ru_1_W_0.14_Zn_1.47_O_x_ catalyst in alkali, suggesting its potential application in future AEMFCs.

## 4. Conclusions

In summary, we have synthesized a series of Ru-W-Zn-O nanocage composites via hydrothermal and annealing treatments using ZIF-8 as the template. The noble metal loading of the Ru_1_W_0.14_Zn_1.47_O_x_ catalyst is only 4.5 wt%, which has greatly reduced the cost of the anode material. Meanwhile, the optimized Ru_1_W_0.14_Zn_1.47_O_x_ nanocage catalyst, with a moderate amount of W, exhibits a high electrochemical surface area of 116 m^2^ g^−1^ and excellent HOR activity, with a mass activity of 0.9 mA µg^−1^ at an overpotential of 50 mV, surpassing that of commercial Pt/C and Ru/C. The high catalytic activity is primarily attributed to the incorporation of W, which modulates the d-band structure of Ru and optimizes the binding ability between active sites and intermediates. Moreover, the three-dimensional hollow porous structure promotes mass transfer and diffusion, accelerating H_2_ transfer and improving the electrocatalytic performance. Our work presents a high-performance Pt-free catalyst material for alkaline HOR and offers valuable insights for developing highly active electrocatalysts.

## Data Availability

The data presented in this study are available on request from the corresponding author.
